# Temporomandibular Disorders Beyond Orofacial Pain: A Narrative Review of Musculoskeletal, Headache, and Central Nervous System Implications

**DOI:** 10.3390/medicina62081546

**Published:** 2026-08-12

**Authors:** Gawon Choe, Ji Hye Hwang

**Affiliations:** 1 Sandol Korean Medicine Clinic, Sejong 30064, Republic of Korea; lovelydoctor@daum.net; 2 Department of Acupuncture and Moxibustion Medicine, Pusan National University, Pusan 50612, Republic of Korea; 3 Department of Acupuncture & Moxibustion Medicine, College of Korean Medicine, Gachon University, Seongnam 13120, Republic of Korea

**Keywords:** bruxism, central sensitization, dental occlusion, postural control, temporomandibular disorders

## Abstract

*Background:* Temporomandibular disorders (TMD), bruxism, and occlusal dysfunction have traditionally been managed as localized orofacial conditions. Emerging evidence suggests, however, that the stomatognathic system may interact with broader neuromusculoskeletal and central pain-processing networks, with potential systemic and neurological implications. *Methods:* A structured narrative literature search was conducted using PubMed, Google Scholar, and Web of Science, with the final targeted search performed in June 2026. Original research articles, systematic reviews, meta-analyses, and relevant pilot studies were considered. No formal risk-of-bias or certainty-of-evidence assessment was performed. *Results:* The reviewed literature indicates that TMD and bruxism are associated with musculoskeletal pain beyond the orofacial region, cervical musculoskeletal dysfunction, and headache comorbidity, with relatively stronger evidence derived from systematic reviews and meta-analyses. Emerging neuroimaging evidence suggests alterations in central pain-modulatory networks, including the default mode network, which may be relevant to central sensitization, although this evidence remains preliminary. Clinically accessible parafunctional signs may prompt further orofacial assessment, and individualized intraoral splint therapy has been investigated for effects beyond local symptom relief, although the evidence remains heterogeneous across domains. *Conclusions:* TMD and occlusal dysfunction may be better understood within a broader neuromusculoskeletal framework. Multidisciplinary assessment and management may warrant consideration in selected patients. Future research should incorporate standardized TMD diagnostic criteria and, where relevant, neuroimaging and posturographic outcomes.

## 1. Introduction

Temporomandibular disorders (TMD) are a heterogeneous group of conditions affecting the temporomandibular joints, masticatory muscles, and associated structures, with manifestations ranging from localized orofacial pain and impaired jaw function to symptoms involving broader musculoskeletal and pain-related domains [1,2]. The Diagnostic Criteria for Temporomandibular Disorders (DC/TMD), developed by Schiffman et al. in 2014, provide a widely used international framework for the clinical and research assessment of TMD [2]. The DC/TMD incorporates standardized physical examination procedures (Axis I) together with assessment of psychosocial status and pain-related disability (Axis II), reflecting the multidimensional nature of TMD. However, heterogeneity in the diagnostic criteria used across the studies reviewed herein may limit direct comparability and should be considered when interpreting the evidence.

Historically, TMD has been approached primarily as a localized dental or maxillofacial problem. However, accumulating evidence suggests that the stomatognathic system may interact with broader systems involved in postural control, musculoskeletal function, and central nervous system processing. Mechanoreceptors in the periodontal ligament and temporomandibular joint provide continuous proprioceptive input to the trigeminal system, which interfaces with vestibular, cerebellar, and spinal pathways involved in postural regulation [3,4]. Alterations in occlusal balance may therefore be associated with changes in cervical alignment, whole-body posture, and pain processing [5,6,7].

Bruxism, a repetitive jaw-muscle activity characterized by clenching or grinding of the teeth and/or by bracing or thrusting of the mandible, represents a potentially relevant behavioral factor in TMD and related musculoskeletal symptoms [8,9]. Associations with tension-type headache, migraine, and musculoskeletal pain beyond the orofacial region have been increasingly documented [10,11,12]. Furthermore, neuroimaging studies have suggested that patients with TMD-related pain may exhibit altered activity or connectivity within the default mode network (DMN), a network implicated in chronic pain-related processing [13,14]. Despite this growing body of evidence, the interrelationships among occlusal dysfunction, postural instability, TMD, headache, and central sensitization remain incompletely understood. Intraoral splint and occlusal interventions have been investigated not only for local pain and muscle activity but also for possible changes in postural parameters [15,16] and cortical organization [17]; however, these multidimensional findings remain heterogeneous and are rarely discussed within a unified framework. Previous reviews have noted associations between temporomandibular dysfunction, spinal alignment, and whole-body posture [18,19], but have generally not integrated emerging central neuroimaging evidence, considered clinically accessible parafunctional signs such as mandibular tori as possible prompts for further assessment, or descriptively compared the relative strength of evidence across these heterogeneous domains.

This narrative review aimed to synthesize current evidence on the systemic and neurological implications of TMD and occlusal dysfunction, spanning postural control, musculoskeletal pain, headache, and central nervous system changes. By presenting an integrated perspective, we sought to highlight the potential clinical relevance of multidisciplinary assessment and management and to identify areas that warrant further research.

## 2. Methods

The literature search was conducted iteratively during the development of this narrative review, with the final targeted search performed in June 2026. Initial thematic literature identification was conducted by G.C., while additional literature searches, relevance screening, and thematic organization were conducted by both authors. The final selection of publications was reviewed and agreed upon by both authors.

PubMed, Google Scholar, and Web of Science were searched using combinations of the following terms: “temporomandibular disorders,” “TMD,” “dental occlusion,” “occlusal proprioception,” “postural control,” “bruxism,” “tension-type headache,” “migraine,” “default mode network,” “central sensitization,” “neuroimaging,” “mandibular torus,” and “intraoral splint.” Boolean operators (AND and OR) were used as appropriate. No restrictions were placed on publication date.

Original research articles, systematic reviews, meta-analyses, and relevant pilot or experimental studies were considered for inclusion. Titles and abstracts were screened for relevance to the neurological, musculoskeletal, postural, and pain-related dimensions of TMD and occlusal dysfunction, followed by full-text assessment of potentially relevant articles. Additional publications were identified by screening the reference lists of retrieved articles. Articles not available in English or not directly relevant to the review objectives were excluded.

**Figure 1:**
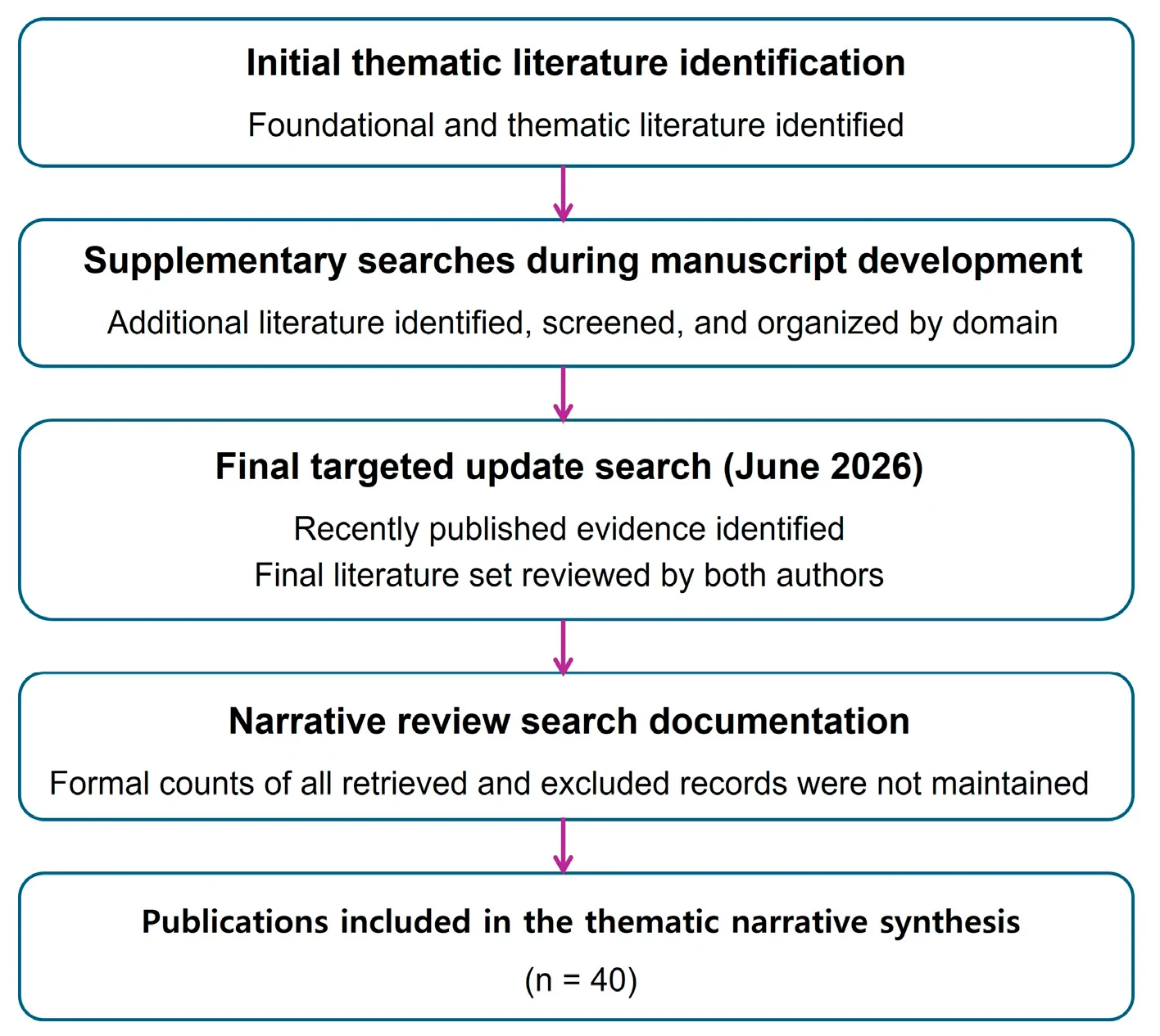
Overview of the iterative literature identification and selection process. Forty publications were included in the six-domain thematic synthesis, excluding references used only for background or contextual discussion. Because complete retrieval and exclusion records were not maintained, the diagram is not PRISMA-compliant.

Because the search was conducted iteratively, a formal record of all initially retrieved and excluded records was not maintained. Forty publications formed the basis of the thematic narrative synthesis. Additional references were used to provide diagnostic background and contextual discussion but were not included in this count. The iterative literature identification and selection process is summarized in Figure 1. The selected literature was organized into six thematic domains: occlusal proprioception and postural control, TMD and musculoskeletal pain, TMD and headache, central pain processing, mandibular tori as potential clinical screening clues, and intraoral splint therapy. No formal risk-of-bias assessment or quantitative meta-analysis was performed, and study selection involved a degree of interpretive judgment inherent to narrative synthesis.

For the descriptive comparison of evidence strength across domains, the relative strength of evidence was summarized according to the predominant study designs within each domain. Findings supported by systematic reviews or meta-analyses were interpreted as relatively stronger, whereas findings derived primarily from observational, pilot, or preliminary neuroimaging studies were regarded as more limited. This categorization was intended as a narrative summary and should not be interpreted as a formal certainty-of-evidence assessment.

## 3. Results

### 3.1. Occlusal Proprioception and Postural Control

The relationship between dental occlusion and whole-body postural control has been the subject of growing scientific interest over the past three decades. The teeth and periodontium are richly innervated by mechanoreceptors that transmit proprioceptive signals via the trigeminal nerve to the brainstem nuclei, which in turn project to the vestibular, cerebellar, and spinal cord pathways involved in postural regulation. This neuroanatomical connectivity forms the theoretical basis for the potential influence of occlusal inputs on body equilibrium [4].

Experimental evidence provides some support for this mechanistic framework. A study conducted on astronauts suggested that wearing an occlusal splint was associated with more symmetrical sternocleidomastoid muscle contraction and reduced body sway, suggesting a possible association between occlusal proprioceptive input and neuromuscular stabilization [15]. Similarly, artificial manipulation of dental occlusion has been reported to be associated with alterations in sensory information flow within the motor system and changes in upper-body postural stabilization, which may involve central nervous system processing [6]. Furthermore, studies involving experimental unilateral alterations of occlusal support have reported immediate changes in head position, posture, and gaze stabilization, consistent with a proposed interplay between occlusion and the neuromusculoskeletal system [18,19]. Clinical studies have further explored these findings, though largely in specialized populations. A pilot study involving professional ballet dancers found that six months of customized occlusal appliance use was associated with improvements in craniofacial and masticatory muscle parameters, as well as balance performance as assessed by the Flamingo Balance Test [16]. A systematic review suggested that malocclusion may be associated with postural balance and that the potential effects of occlusal interventions on postural stability warrant further investigation [7].

Much of the evidence in this domain is based on small experimental or pilot studies, often conducted in specialized populations such as athletes or astronauts. Causal directionality and generalizability to broader clinical populations have yet to be established through larger controlled trials. A recent scoping review further indicated that the relationship between occlusion and TMD remains complex and debated, suggesting that occlusal findings should be interpreted within a broader biopsychosocial and neuromusculoskeletal framework, rather than as a single deterministic cause of TMD [20].

### 3.2. TMD, Bruxism, and Musculoskeletal Pain

TMD have long been recognized as a source of orofacial pain; however, emerging evidence indicates that their clinical impact may extend considerably beyond the craniofacial region. Epidemiological studies have suggested that individuals with TMD are significantly more likely to report pain in other body regions than unaffected individuals. A cross-sectional study reported that individuals with TMD were approximately 5.5 times more likely to report pain in other joints, with particularly strong associations observed for knee pain, low back pain, and nonspecific musculoskeletal complaints [10]. These findings suggest the possible involvement of shared mechanisms, including central sensitization, in the co-occurrence of TMD and widespread pain conditions.

Bruxism, encompassing both sleep and awake bruxism, is an important behavioral factor associated with TMD. A systematic review and meta-analysis reported that the presence of bruxism was associated with approximately 2.25-fold higher odds of TMD (odds ratio [OR], 2.25; 95% confidence interval [CI], 1.94–2.56), indicating a substantial association between the two conditions [9]. Proposed mechanisms underlying this association include sustained masticatory muscle overactivity, microtrauma to the temporomandibular joint, and altered pain thresholds associated with chronic muscle fatigue.

The clinical relevance of bruxism is further underscored by recent systematic reviews and meta-analytic evidence indicating that both sleep and awake bruxism are prevalent across pediatric and adult populations, although prevalence estimates vary substantially according to diagnostic criteria and assessment methods [21]. Increased masseter muscle thickness may represent a clinically observable manifestation associated with sustained parafunctional activity. Patients with concurrent TMD and bruxism have been reported to exhibit greater masseter muscle thickness than TMD patients without bruxism, as assessed by ultrasonography, with bruxism reported as an independent statistical predictor of muscle thickness [22]. Greater mechanosensitivity of the masticatory muscles has also been observed in patients with myofascial TMD and bruxism, suggesting a possible association between parafunctional muscle activity and local or referred pain [23]. However, increased masseter thickness should not be regarded as a definitive diagnostic marker for either bruxism or TMD, because it may occur in the absence of joint pathology and its diagnostic specificity remains limited. Its identification during clinical examination may nevertheless prompt further assessment for bruxism and, where appropriate, evaluation for TMD using established diagnostic criteria such as the DC/TMD [2].

The cervical spine represents another important domain of broader musculoskeletal involvement associated with TMD. A systematic review and meta-analysis found that patients with TMD exhibited significantly reduced cervical range of motion, diminished cervical flexor muscle endurance, and greater self-reported neck disability compared with healthy controls [24]. This cervical musculoskeletal dysfunction may reflect both the anatomical continuity of the trigeminal and cervical nociceptive systems (the trigeminocervical complex) and postural adaptations associated with chronic orofacial pain [3]. Collectively, these findings suggest that TMD should not be viewed solely as an isolated local condition. Assessment of cervical and peripheral musculoskeletal function may warrant consideration in patients with TMD, while evaluation for possible TMD or bruxism may be relevant in selected patients presenting with widespread pain.

### 3.3. TMD, Bruxism, and Headache

Headache is one of the most frequently reported comorbidities in patients with TMD and bruxism. A systematic review found that awake bruxism was associated with 5- to 17-fold higher odds of tension-type headache (across studies, low to very low certainty), whereas the association with migraine was inconsistent, reaching significance in some but not all studies [12]. These figures suggest that bruxism-related masticatory muscle overactivity may be involved in the co-occurrence or persistence of primary headache disorders, potentially through sustained pericranial muscle tension and sensitization of trigeminal afferents. The overlap between TMD and headache was further supported by prevalence data. In a single cross-sectional study (*n* = 96), the self-reported prevalence of headache among patients with painful TMD was approximately 82.8%, substantially higher than that reported in the general population. Importantly, the study reported that this association was partially influenced by bruxism and somatic symptom burden, suggesting that the relationship between TMD and headache may involve shared psychophysiological vulnerability factors rather than purely mechanical mechanisms [11].

From a neurobiological perspective, the convergence of trigeminal and cervical nociceptive inputs at the level of the trigeminal nucleus caudalis provides a plausible anatomical substrate for the co-occurrence of orofacial pain and headache. Peripheral sensitization of masticatory and pericranial muscles, combined with central sensitization associated with persistent nociceptive input, may lower the threshold for headache occurrence in susceptible individuals [25]. More recent systematic reviews and meta-analyses have further supported the clinical overlap between TMD and primary headache disorders, including migraine and tension-type headache, suggesting the potential value of reciprocal screening between headache and orofacial pain populations [26]. Clinically, the frequent co-occurrence of TMD, bruxism, and headache underscores the importance of interdisciplinary evaluation. Patients presenting with chronic or recurrent headache may benefit from orofacial assessment, while patients with TMD reporting headache should be evaluated according to established headache classification criteria, such as those outlined in the International Classification of Headache Disorders, third edition (ICHD-3) [27].

### 3.4. Central Pain Processing and the Default Mode Network

Beyond its musculoskeletal and headache-related manifestations, TMD may also be associated with alterations in central nervous system processing. Advances in neuroimaging, particularly functional magnetic resonance imaging (fMRI), have provided preliminary evidence that chronic orofacial pain and dysfunction may be accompanied by differences in brain activation patterns, suggesting a neurological dimension of TMD that warrants further investigation.

The DMN, a set of interconnected brain regions that includes the medial prefrontal cortex, posterior cingulate cortex, and precuneus, is involved in self-referential processing and has been implicated in the affective and cognitive dimensions of chronic pain. Preliminary studies have suggested that patients with TMD-related pain may exhibit reduced activation of key DMN regions during tooth-clenching tasks compared with healthy controls, a pattern that may be associated with differences in endogenous pain modulation and pain sensitivity; however, this finding was derived from a single small functional MRI study (*n* = 36) and requires replication [14].

Complementary fMRI evidence has identified differences in cortical activation patterns during tooth clenching in patients with TMD, including reduced activity in motor and cognitive processing regions, suggesting that chronic orofacial dysfunction may be associated with broader differences in cortical network organization [13]. These findings are consistent with the broader literature on central sensitization in chronic pain conditions, in which sustained peripheral nociceptive input may be associated with maladaptive changes in central pain processing [5].

A single-subject fMRI study observed changes in cortical activation patterns following three months of occlusal splint therapy, including increased activation of parietal sensorimotor integration areas [17]. While the very limited sample size of this study precludes definitive conclusions, these preliminary observations raise the possibility that occlusal intervention may be associated with neuroplastic changes, a hypothesis requiring investigation in larger controlled neuroimaging studies. Recent reviews have further described the potential roles of peripheral sensitization, central sensitization, and neuroplastic changes within the trigeminal system in the persistence of chronic TMD-related pain [28]. Another review highlighted the potential utility of functional neuroimaging, including BOLD-fMRI, for investigating central pain mechanisms in chronic painful TMD and discussed the emerging application of artificial intelligence in integrating neuroimaging data [29].

In addition to neuroimaging findings, recent prospective observational evidence has highlighted the prevalence of central sensitization and somatization among adults with TMD, suggesting that altered central pain processing may be clinically relevant in this population [30]. Taken together, these emerging findings suggest that TMD may have central neurological correlates beyond peripheral tissue involvement. This perspective, although still supported by limited evidence, may have implications for understanding chronic pain mechanisms in this population. The proposed pathways linking stomatognathic dysfunction with central nervous system alterations are summarized in Table 1.

### 3.5. Clinical Screening Clues: Mandibular Torus and Parafunctional Signs

Mandibular tori, benign bony exostoses located on the lingual aspect of the mandible, have traditionally been regarded as incidental anatomical findings of limited clinical significance. However, emerging evidence suggests that their presence may represent a clinically accessible clue associated with chronic occlusal loading and masticatory muscle hyperactivity, with potential relevance to the assessment of bruxism and TMD [31,32,33,34]. The association between mandibular tori and bite force has been explored quantitatively. Quantitative evaluation has suggested that torus size may increase proportionally with occlusal force, which is consistent with the hypothesis that tori may develop as an adaptive osseous response to sustained mechanical loading of the mandible [31]. This reported association suggests that the identification of mandibular tori during clinical examination may provide a possible low-cost clue to habitual parafunctional activity. The potential clinical relevance of this observation has also been examined in populations with TMD and headache. Mandibular tori have been reported to be significantly more prevalent among patients attending facial pain clinics with diagnoses of migraine and TMD compared to general dental patients [32]. Furthermore, a systematic review identified a significant association between signs and symptoms of bruxism and the presence of oral tori [33]. Specific correlations have also been observed between mandibular tori and certain subtypes of TMD [34].

Although these findings are promising, mandibular tori should not be characterized as a validated biomarker for TMD or bruxism. Rather, their identification during routine clinical examinations may serve as one of several potential screening clues, alongside tooth wear, masticatory muscle tenderness, and cervical dysfunction. These signs should be regarded as prompts for further evaluation rather than as diagnostic markers, and formal TMD assessment should rely on established diagnostic criteria. Validation in larger prospective cohorts is required before clinical recommendations can be firmly established.

### 3.6. Intraoral Splint Therapy: Local and Potential Systemic Effects

Intraoral splint therapy represents the most widely employed conservative intervention for TMD and bruxism, with an evidence base supporting possible benefits in reducing orofacial pain and masticatory muscle hyperactivity [35]. Beyond local symptom management, some studies have suggested that occlusal splint use may be associated with changes in postural and neuromuscular domains, although the strength of this evidence varies considerably [36,37,38,39,40,41,42]. Recent evidence from systematic reviews suggests that occlusal splint therapy may provide benefits for selected TMD-related outcomes, particularly pain and functional symptoms, although the magnitude of effect, comparator conditions, splint design, and patient characteristics vary substantially across studies [35]. Therefore, evidence supporting local symptom management should be distinguished from the more preliminary evidence regarding systemic, postural, or neurological effects.

The foundational rationale for possible broader effects of splint therapy derives from early neuromuscular models, which proposed that customized mandibular orthopedic repositioning appliances (MORA) may improve neuromuscular efficiency, with associated benefits in muscle strength, body coordination, and craniocervical postural alignment [36]. Subsequent research has reported improvements in muscular endurance and anaerobic power associated with a neuromuscular dentistry-designed mouthguard in trained individuals [37]. Recent controlled studies have provided additional support for these systemic effects. A randomized controlled trial reported that the combination of occlusal splint therapy and therapeutic exercises was associated with significant improvements in postural balance in patients with TMD signs and symptoms, as measured by force-platform analysis [38]. Furthermore, a prospective case–control study reported significant craniovertebral and craniomandibular changes following six months of occlusal splint therapy combined with physiotherapy, including alterations in the functional space between C1 and C2 and changes in cervical posture, as assessed by lateral cephalometry [39].

However, the evidence is not uniformly positive. Studies employing over-the-counter (OTC) jaw-repositioning devices have yielded inconsistent results, finding no significant improvements in dynamic balance, flexibility, agility, strength, or power in college-aged male athletes using OTC mouthguards [40]. This discrepancy between customized and OTC devices suggests that individualized mandibular positioning may influence treatment-related outcomes, although this hypothesis requires further controlled investigation. Conversely, recent prospective clinical evidence suggests that the effect of occlusal stabilization splints on global body posture may be limited, with changes more likely to be observed in selected cervicocranial parameters than in whole-body postural alignment [41]. Moreover, systematic reviews have reported possible benefits of specific anterior bite planes, such as the nociceptive trigeminal inhibition tension suppression system (NTI-tss), for bruxism and TMD management; however, these devices do not provide full-arch stabilization [42].

The neurological observations described in Section 3.4, including preliminary evidence of cortical changes following splint therapy, further suggest that the potential implications of occlusal intervention may extend beyond local symptom relief. Taken together, the available evidence suggests that appropriately designed intraoral splints may be associated with effects extending beyond local symptom relief, although the magnitude and clinical significance of findings across postural, neuromuscular, and neurological domains remain to be established through larger controlled trials. The key studies supporting the evidence reviewed in this article are summarized in Table 2. The relative strengths of the evidence across all reviewed domains are presented in Table 3.

**Table 2:** Summary of key included studies supporting the evidence domains reviewed.

Author (Year)	Study Design	Subjects (*n*)	Key Findings	Domain
Stancker et al. [7]	Systematic review	Multiple studies	Malocclusion may influence postural balance; occlusal interventions may improve postural stability	Occlusion & posture
Bonato et al. [10]	Cross-sectional study	*n* = 337	Individuals with TMD ~5.5× more likely to report pain in other joints; associations with knee and low back pain	Systemic pain
Mortazavi et al. [9]	SR & meta-analysis	Multiple studies	Bruxism associated with ~2.25× higher odds of TMD development (OR 2.25, 95% CI 1.94–2.56)	Bruxism & TMD
de Oliveira-Souza et al. [24]	SR & meta-analysis	Multiple studies	TMD patients show reduced cervical ROM, diminished flexor endurance, greater neck disability	Cervical dysfunction
Réus et al. [12]	Systematic review	Multiple studies	Awake bruxism associated with 5–17× higher odds of tension-type headache (no meta-analysis; representative OR 5.23, 95% CI 2.57–10.65; low/very low certainty)	TMD & headache
van der Meer et al. [11]	Cross-sectional study	*n* = 96	Headache prevalence is 82.8% in painful TMD; association partially confounded by bruxism and somatic symptoms	TMD & headache
Bizzarri et al. [26]	SR & meta-analysis	Multiple studies	Significant clinical overlap between TMD and primary headache disorders including migraine and TTH	TMD & headache
Kluskens et al. [14]	fMRI study	*n* = 36	TMD patients show reduced DMN activation during clenching; associated with impaired pain inhibition	Central pain/DMN
Seweryn et al. [30]	Prospective observational	*n* = 214	High prevalence of central sensitization and somatization among adults with TMD	Central pain/DMN
Oliveira et al. [38]	RCT	*n* = 40	Occlusal splint + therapeutic exercises significantly improved postural balance (force platform)	Splint therapy
Derwich et al. [39]	Prospective case–control	*n* = 50	6 months of splint + physiotherapy led to significant craniovertebral and C1–C2 changes	Splint therapy
Del Sorbo et al. [35]	Umbrella review	21 systematic reviews	Majority of SRs report small beneficial or neutral effects of occlusal splints on TMD outcomes	Splint therapy
Hampe et al. [41]	Prospective clinical trial	*n* = 24	Influence of stabilization splints on global body posture is limited; small effects on cervicocranial parameters only	Splint therapy

Abbreviations: DMN, default mode network; fMRI, functional magnetic resonance imaging; RCT, randomized controlled trial; ROM, range of motion; ; SR, systematic review; TTH, tension-type headache; TMD, temporomandibular disorders.

**Table 3:** Summary of key evidence domains, main study designs, key findings, evidence strength, and clinical interpretation.

Domain	Main Evidence	Key Findings	Strength	Interpretation
Occlusion and postural control	Experimental studies, pilot studies, scoping/systematic reviews	Occlusal input may affect postural sway and balance	Low-moderate	Mechanistically plausible but debated; heterogeneous samples
TMD and systemic pain	Systematic review/meta-analysis	TMD associated with pain in other joints and widespread pain (OR ~5.5)	Moderate	Association relatively consistent across studies
Bruxism and TMD	Systematic review/meta-analysis	Bruxism associated with increased TMD odds (OR ~2.25)	Low-moderate	Behavioral association; bidirectional relationship possible
TMD/bruxism and headache	Systematic reviews, clinical studies	Tension-type headache and migraine associations (5–17× odds; migraine inconsistent)	Low-moderate	Relatively consistent clinical overlap; targeted assessment may be considered in selected patients.
TMD and DMN/CNS	fMRI studies, small samples	Altered activation in pain-related brain networks including DMN	Low	Emerging central pain processing evidence; replication needed
Mandibular torus	Observational studies, systematic review	Associated with bite force, bruxism signs, and TMD	Low-moderate	Potential screening clue; not yet validated as biomarker
Splint therapy	Clinical, pilot, and intervention studies	Possible local, postural, and neuromuscular effects	Low-moderate	Modest local benefits in selected outcomes; systemic/postural effects remain preliminary

Evidence strength was informally assessed based on the overall consistency, study design, and limitations of the available literature: moderate, supported by systematic review or meta-analytic evidence with relatively consistent findings; low-moderate, supported by systematic reviews, controlled studies, or multiple observational studies with substantial heterogeneity or important methodological limitations; and low, based primarily on small pilot studies, single case studies, or preliminary neuroimaging evidence. Formal evidence grading (e.g., GRADE) was not applied given the narrative nature of this review. Abbreviations: DMN, default mode network; TMD, temporomandibular disorder.

## 4. Discussion

The evidence synthesized in this review collectively suggests a model in which the stomatognathic system functions as an integral node within a broader network encompassing postural control, musculoskeletal pain, headache generation, and central nervous system regulation. This interconnectedness has been conceptualized in the “cybernetic unit” model, which proposes that the temporomandibular and craniocervical joints may function as a regulated unit influenced by neurological and neurophysiological reflexes [43]. This systemic perspective is also consistent with review literature suggesting associations between temporomandibular dysfunction, spinal alignment, and whole-body postural adaptations [44]. Rather than a purely localized orofacial condition, TMD and its associated phenomena (occlusal dysfunction, bruxism, and masticatory muscle hyperactivity) appear from this body of literature to have broader neuromusculoskeletal dimensions with potential clinical implications [9,10,12,24,43,44].

A unifying mechanistic thread running through the reviewed evidence is the role of trigeminal proprioception as a key interface between the stomatognathic system and the rest of the body (Section 3.1). Alterations in periodontal and temporomandibular joint proprioceptive input, whether associated with malocclusion, bruxism-related joint loading, or masticatory muscle dysfunction, may be associated with postural instability and cervical musculoskeletal impairment documented across the reviewed studies [3,4,7]. Separately, persistent nociceptive input may also be associated with widespread pain sensitization and changes in central pain processing [5,24]. Central sensitization therefore emerges as a second proposed mechanism linking the reviewed domains. Chronic nociceptive input from the orofacial region may be associated with changes at the level of the trigeminal nucleus caudalis and higher cortical centers [3,5,28,29]. The neuroimaging findings reviewed in Section 3.4—including possible DMN hypoactivation and altered cortical recruitment during clenching tasks—may represent central nervous system correlates of this sensitization process. These findings may reflect altered central pain processing, although their relationship with widespread symptoms and comorbidities remains uncertain [13,14,30].

**Figure 2:**
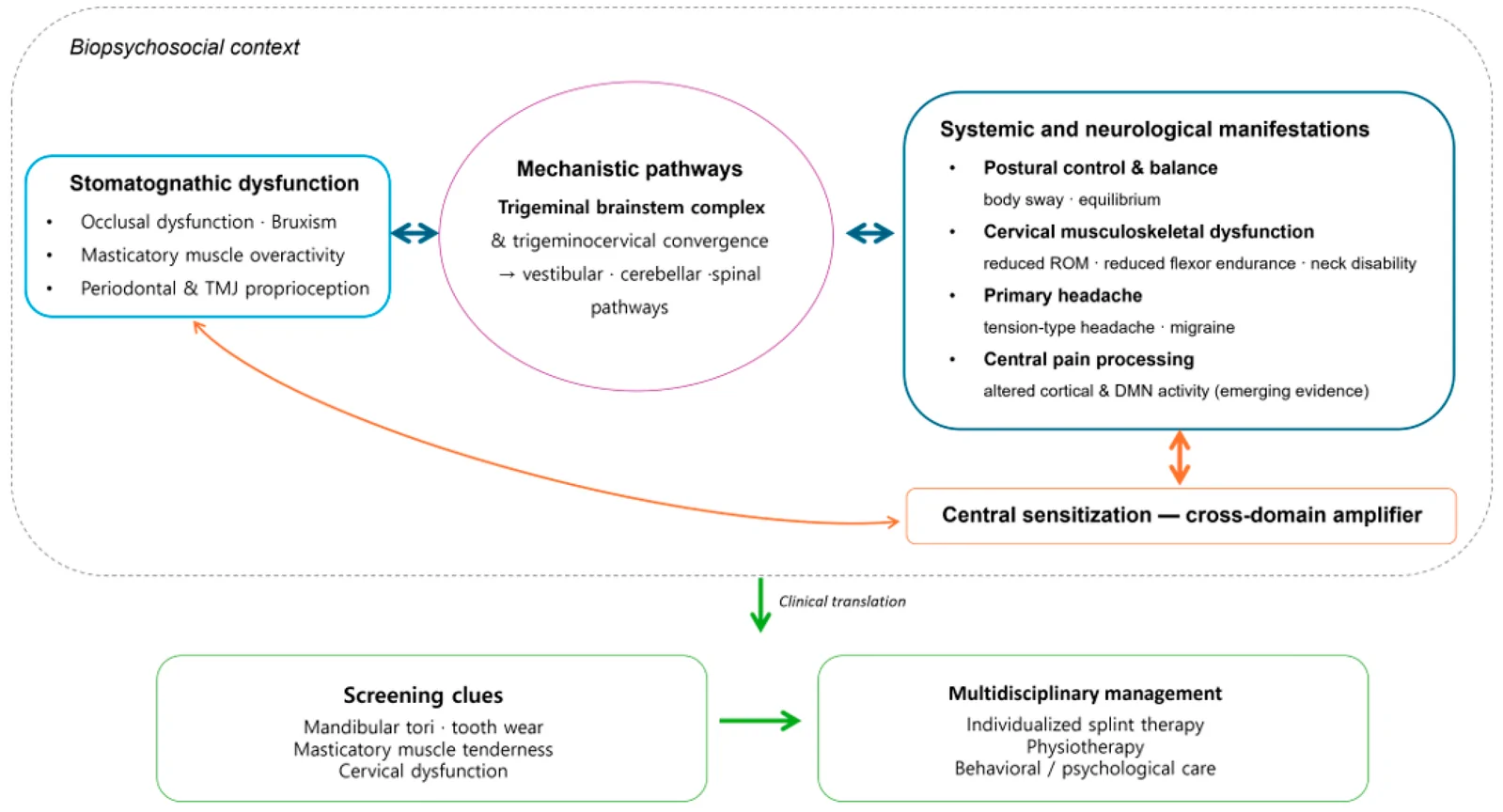
Proposed hypothetical neuromusculoskeletal framework linking temporomandibular disorders (TMD), bruxism, and occlusal dysfunction with broader musculoskeletal and neurological manifestations. Periodontal and temporomandibular joint proprioceptive input, together with bruxism-associated masticatory muscle overactivity, may be conveyed through the trigeminal brainstem complex and trigeminocervical convergence, which interface with vestibular, cerebellar, and spinal pathways. These interactions may be associated with four interrelated domains—postural control and balance, cervical musculoskeletal dysfunction, primary headache, including tension-type headache and migraine, and central pain processing, including altered cortical and default mode network (DMN) activity—with central sensitization proposed as a shared cross-domain amplifier. Double-headed arrows denote proposed associations and bidirectional interactions rather than established unidirectional causal pathways. The framework is intended to operate within and complement, rather than replace, the biopsychosocial model of TMD. Potential clinical implications include targeted orofacial assessment and multidisciplinary management in selected patients, with individualized splint therapy considered where appropriate. Abbreviations: DMN, default mode network.

The evidence reviewed in this article should not be interpreted as uniformly strong across all domains. The associations among TMD, bruxism, headache, and cervical musculoskeletal dysfunction have received support from systematic reviews and meta-analyses [9,10,12,24,26], although important heterogeneity remains. In contrast, evidence linking occlusal modulation to whole-body postural regulation and central network reorganization remains preliminary and often relies on small experimental or pilot studies [15,16,17,41]. Therefore, the proposed framework should be interpreted as an integrative and hypothesis-generating model rather than a definitive causal pathway. The key evidence domains and their relative strengths are listed in Table 3, and the proposed integrated framework is illustrated in Figure 2. Importantly, this framework should complement, rather than replace, the biopsychosocial model of TMD, as psychological factors, behavioral patterns, sleep disturbances, and somatic symptom burden may substantially influence symptom expression and treatment responses.

The clinical implications of this integrated model may be relevant, although further validation is required. Targeted orofacial screening in selected patients presenting with postural complaints, cervical musculoskeletal dysfunction, or chronic headache may be considered, because coexisting TMD or parafunctional activity may otherwise remain unrecognized. Masticatory muscle tenderness and clinically evident tooth wear may prompt further assessment for TMD or parafunctional activity [2,8]. Mandibular tori have also been reported in association with parafunctional loading, although their diagnostic value as a screening clue remains uncertain [31,33]. A multidisciplinary approach to TMD management, in which dental, medical, and allied health professionals collaborate in assessment and treatment planning, may be considered in selected patients, given the breadth of associations reported in the literature. A recent narrative review also described the potential value of multimodal approaches, including physiotherapy, occlusal splints, behavioral interventions, and psychotherapy, for symptom management in patients with TMD [45]. Botulinum toxin injections have also been investigated for bruxism-associated masticatory muscle hyperactivity and masseter hypertrophy [46,47]. However, because the present review was not designed to comprehensively evaluate treatment efficacy, botulinum toxin is mentioned only briefly as a therapeutic approach of interest. Its indications, dosing, long-term effectiveness, and safety require separate systematic evaluation.

The integrated framework synthesized in this review may also provide a conceptual context for temporomandibular balancing-based approaches. These approaches may be viewed as attempting to integrate several of the domains discussed above, including temporomandibular and periodontal proprioception, trigeminal and trigeminocervical pathways, postural regulation, bruxism-related musculoskeletal loading, and central pain processing. They should not, however, be regarded as independent mechanistic models or as clinically validated applications of these emerging associations. The available clinical evidence for temporomandibular balancing therapy (TBT) remains limited. A published case-based report has described its use as part of Korean medicine–based integrative treatment for complex neuromusculoskeletal symptoms [48]; however, such reports constitute preliminary clinical descriptions rather than direct evidence of efficacy. A recently published randomized controlled trial protocol comparing TBT with transcutaneous electrical nerve stimulation in patients with chronic TMD represents an initial step toward its direct clinical evaluation, with outcomes including pain intensity, jaw function, and cost-effectiveness [49]. The mechanistic framework presented in this review may provide a conceptual basis for interpreting future clinical findings, while premature conclusions regarding the efficacy or underlying mechanisms of TBT should be avoided. Its clinical indications, effectiveness, and underlying mechanisms remain to be established through adequately powered controlled trials.

Several limitations of the current evidence base warrant further investigation. Mechanistic studies linking occlusal proprioception to postural control are largely based on small samples, often from specialized populations. The causal directionality of many of the observed associations remains incompletely established, and reverse causality cannot be excluded. Although conceptually compelling, the neuroimaging literature on TMD and DMN dysfunction is limited to a small number of studies with restricted sample sizes. Furthermore, the heterogeneity of splint designs, outcome measures, and patient populations across intervention studies complicates direct comparisons and limits the generalizability of conclusions. In particular, recent prospective evidence suggests that the influence of occlusal stabilization splints on global body posture may be limited, with effects more likely to be confined to cervicocranial parameters, supporting cautious interpretation of splint-related postural effects and consideration of multimodal management in selected patients [41]. Future research should prioritize prospective longitudinal studies examining the natural history of central sensitization in TMD populations, alongside controlled trials evaluating the neurological and postural effects of individualized occlusal intervention. The integration of neuroimaging, electromyography, and posturographic outcome measures within unified study designs would substantially advance the mechanistic understanding.

In conclusion, this narrative review suggests that TMD, bruxism, and occlusal dysfunction may be considered beyond their traditional characterization as purely localized orofacial conditions. The synthesized literature supports a model in which the stomatognathic system may function as an interconnected component within a broader neuromusculoskeletal network linked to cervical biomechanics, whole-body postural control, and central pain processing. Trigeminal proprioceptive pathways, trigeminocervical convergence, and central sensitization mechanisms may provide neurobiological substrates for these diverse systemic manifestations; however, the causal relationships and directionality of these associations require further investigation. From a clinical perspective, these findings suggest the potential value of interdisciplinary collaboration. In selected patients, the identification of parafunctional signs, including tooth wear, masticatory muscle tenderness, and mandibular tori, may prompt further orofacial assessment, particularly in the context of complex musculoskeletal complaints or chronic headaches. Individualized intraoral interventions may be considered within multidisciplinary care, with preliminary evidence suggesting possible effects on selected postural and pain-processing outcomes, whereas the systemic and neurological effects of temporomandibular balancing-based approaches remain to be established through controlled trials. Future research should prioritize controlled studies incorporating standardized TMD diagnostic criteria, functional neuroimaging, posturographic assessments, and patient-reported outcomes to clarify causal mechanisms and support the development of evidence-based multidisciplinary management strategies.

## 5. Conclusions

This narrative review suggests that TMD, bruxism, and occlusal dysfunction may extend beyond localized orofacial disorders and interact with broader neuromusculoskeletal and pain-processing networks. Trigeminal proprioception, trigeminocervical convergence, cervical biomechanics, and central sensitization may contribute to the observed associations with headache, cervical dysfunction, postural alterations, and widespread pain. However, the strength of evidence varies across these domains, and causality remains uncertain.

Targeted orofacial assessment and multidisciplinary management may therefore be considered in selected patients with chronic headache or complex musculoskeletal symptoms. Further prospective and controlled studies using standardized TMD diagnostic criteria and integrated neuroimaging, electromyographic, posturographic, and patient-reported outcomes are needed to clarify these relationships and guide evidence-based care.

## Figures and Tables

**Table 1:** Pathophysiological and clinical links between stomatognathic dysfunction and systemic or central nervous system manifestations.

Clinical Domain	Clinical Manifestations	Proposed Underlying Mechanisms	Key Evidence/Highlights
Musculoskeletal Pain	Widespread pain (e.g., knee, lower back), reduced cervical range of motion, diminished cervical flexor endurance, neck disability	Sustained masticatory muscle overactivity; anatomical continuity via the trigeminocervical complex; postural adaptations	TMD patients are ~5.5 times more likely to report pain in other joints. Bruxism is associated with a ~2.25-fold increased odds of TMD (OR 2.25, 95% CI 1.94–2.56).
Headache Comorbidity	Tension-type headaches, migraines, sustained pericranial muscle tension	Convergence of trigeminal and cervical nociceptive input at the trigeminal nucleus caudalis; peripheral and central sensitization	Awake bruxism is associated with 5- to 17-fold higher odds of tension-type headache (across studies; low to very low certainty). 82.8% self-reported headache prevalence in painful TMD (single cross-sectional study, *n* = 96).
Central Nervous System & DMN	Amplified pain experience, heightened pain sensitivity, altered cognitive and motor processing	Maladaptive neuroplastic changes; hypoactivation of the Default Mode Network (DMN) and altered cortical recruitment during clenching	Reduced activation in key DMN regions is associated with impaired endogenous pain inhibition. Cortical activation patterns may partially normalize following intraoral splint therapy.

Abbreviations: DMN, default mode network; TMD, temporomandibular disorders.

## Data Availability

Data sharing is not applicable to this article as no new data were created or analyzed in this study.

## Abbreviations

The following abbreviations are used in this manuscript:

TMD	Temporomandibular disorders
DMN	default mode network
fMRI	functional magnetic resonance imaging
MORA	mandibular orthopedic repositioning appliances
OTC	over-the-counter
